# Point Defect Scattering and Phonon Softening for Achieving High Thermoelectric Performance in p-Type ZnSb with Optimal Carrier Concentration

**DOI:** 10.1021/acsami.4c21670

**Published:** 2025-03-10

**Authors:** Dongyi Shen, Siu Ting Tai, Kejia Liu, Wenxuan Wang, Haiqi Li, Vaskuri C. S. Theja, Chen Chen, Yue Chen

**Affiliations:** † Department of Mechanical Engineering, The University of Hong Kong, Pokfulam Road, Hong Kong SAR , China; ‡ School of Physical Sciences, Great Bay University, Dongguan, Guangdong 523000, China

**Keywords:** thermoelectrics, zinc antimonide, point defect scattering, phonon softening, carrier concentration optimization

## Abstract

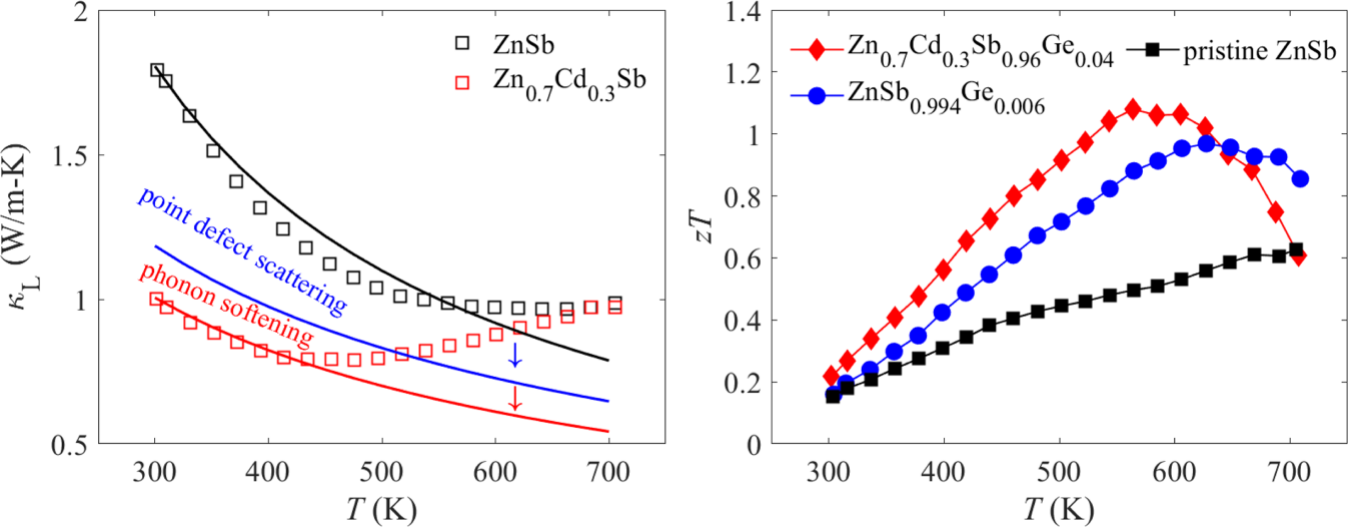

The thermoelectric material ZnSb has been intensively studied on account of its good thermodynamic stability and earth-abundant constituent elements, both of which make it feasible for mass production. However, the practical application of ZnSb is limited by its relatively poor thermoelectric performance, characterized by a low power factor and high lattice thermal conductivity. Herein, we demonstrate that there is a significant improvement in the thermoelectric figure of merit of ZnSb by combining Ge doping at the Sb site with Cd alloying at the Zn site. First, Ge doping at the Sb site can effectively optimize the carrier concentration, thereby resulting in an ∼82% increase in the peak power factor through a concentration of only 0.6%. Second, Cd alloying at the Zn site can bring about a strong point defect scattering to phonon propagation, leading to reduced phonon relaxation time. Meanwhile, the significant softening of acoustic phonons is also introduced by Cd alloying at the Zn site, and thus group velocities of acoustic phonon modes are suppressed. Consequently, a ∼44% reduction in the lattice thermal conductivity is achieved in Zn_0.7_Cd_0.3_Sb at room temperature. As a result of the optimized carrier concentration and suppressed lattice thermal conductivity, a peak *zT* value as high as ∼1.08 at 564 K is attained in Zn_0.7_Cd_0.3_Sb_0.96_Ge_0.04_.

## Introduction

Global energy challenges and climate change lead to extensive research into thermoelectric (TE) materials, which are capable of recovering waste heat into electrical power without greenhouse gas emissions.^1−4^ The energy conversion efficiency of TE materials depends on the dimensionless figure of merit *zT* = *S*^2^σ*T*/κ, where *S* is the Seebeck coefficient, σ is the electrical conductivity, *T* is the absolute temperature, and κ is the total thermal conductivity generally comprising the electronic contribution κ_e_ and the lattice component κ_L_.^5−7^ The quantity *S*^2^σ is usually referred to as the power factor, which is associated with electrical transport properties. To achieve high-efficiency TE materials, we need to either enhance the power factor or suppress the thermal conductivity. Strategies for improving the power factor include carrier concentration optimization,^8,9^ manipulation of carrier scattering mechanism,^10,11^ and band structure engineering.^12,13^ Efforts to reduce the thermal conductivity are mainly focused on decreasing the lattice component via phonon engineering because it is relatively independent of other thermoelectric parameters.^14,15^ In recent years, high *zT* values have been discovered in chalcogenides,^16−18^ half-Heusler alloys,^19,20^ as well as Zintl-phase compounds^21−23^ in consequence of relentless optimization.

In addition to the aforementioned high-performance TE materials, zinc antimonides, typified by β-Zn_4_Sb_3_ and ZnSb, also attract much research attention because constituent elements are earth-abundant and relatively cheap. It is found that β-Zn_4_Sb_3_ has an intrinsically low lattice thermal conductivity of ∼0.65 W m^–1^ K^–1^ at room temperature, which primarily arises from its complex crystal structure with disordered Zn atoms distributed among multiple interstitial sites.^24−27^ High *zT* values above unity are achieved in p-type β-Zn_4_Sb_3_ after optimizing the carrier concentration by introducing various dopants.^28−30^ However, the real-world application of β-Zn_4_Sb_3_ is severely impeded by its poor thermal stability under an electric field or a temperature gradient.^24,26,29^ In contrast, ZnSb is much more stable than β-Zn_4_Sb_3_ when an electric field or a temperature gradient is applied. This is because all Zn atoms in ZnSb are orderly located at specific lattice sites.^24^ From the perspective of thermodynamic stability, ZnSb is expected to be a promising alternative to β-Zn_4_Sb_3_, and many approaches are proposed to improve the TE performance of ZnSb.^24,31−35^ Based on the density functional theory (DFT) calculations, it was predicted that substitution of Sb with Ge can effectively optimize the carrier concentration and thus enhance the *zT* value of ZnSb.^36^ Since there is barely any experimental work to verify this theoretical prediction, it is necessary to explore the effect of Ge doping at the Sb site on the TE performance of ZnSb. Owing to the lack of interstitial Zn atoms, ZnSb has a higher room-temperature lattice thermal conductivity than β-Zn_4_Sb_3_. Although Cd alloying at the Zn site is commonly used to reduce the lattice thermal conductivity, its impact on the phonon propagation is seldom studied.^37−40^

In this work, we demonstrate that high TE performance in p-type ZnSb can be achieved through a combination of optimized carrier concentration and suppressed lattice thermal conductivity. First of all, Ge doping at the Sb site has been proven to be successful at optimizing the carrier concentration. Accordingly, the maximum power factor is remarkably improved from 11 μW cm^–1^ K^–2^ for pristine ZnSb at 705 K to 20 μW cm^–1^ K^–2^ for ZnSb_0.994_Ge_0.006_ at 480 K. Moreover, Cd alloying at the Zn site can dramatically suppress the lattice thermal conductivity to 1 W m^–1^ K^–1^ for Zn_0.7_Cd_0.3_Sb from 1.79 W m^–1^ K^–1^ for pristine ZnSb at room temperature, which stems from point defect scattering and phonon softening. By combining Ge doping at the Sb site and Cd alloying at the Zn site together, a peak *zT* value as high as ∼1.08 is attained in Zn_0.7_Cd_0.3_Sb_0.96_Ge_0.04_ at 564 K due to simultaneous optimization of the electrical and thermal transport properties. This suggests that ZnSb is a potential high-performance TE material.

## Experimental Section

### Sample Synthesis

According to the nominal compositions ZnSb_1–*x*_Ge*_x_* (*x* = 0, 0.001, 0.002, 0.004, and 0.006) and Zn_0.7_Cd_0.3_Sb_1–*y*_Ge*_y_* (*y* = 0, 0.01, 0.02, 0.04, and 0.06), stoichiometric amounts of constituent elements zinc (Zn, shots, 99.999%), antimony (Sb, shots, 99.99%), cadmium (Cd, shots, 99.999%), and germanium (Ge, pellets, 99.9999%) were weighed out and sealed in quartz tubes under vacuum. The quartz tubes were then heated to 1023 K and held at this temperature for 24 h followed by quenching in water. Subsequently, the quenched ingots were annealed at 573 K for 48 h before ball milling into fine powders. Finally, the fine powders were consolidated into dense pellets by hot pressing at 693 K for 5 min under a uniaxial pressure of 50 MPa.

### Sample Characterization

The crystal structure was characterized on an X-ray diffractometer (Rigaku MiniFlex 600-C) with Co K_α_ radiation (λ = 1.7890 Å). The backscattered electron (BSE) imaging and elemental mapping were performed on the field emission scanning electron microscope (FESEM, LEO 1530) equipped with an energy-dispersive X-ray spectrometer (EDS). The measurement of electrical transport properties was carried out in the low-pressure helium atmosphere. The electrical conductivity σ was measured using the van der Pauw technique.^41,42^ The Hall coefficient *R*_H_ was determined by the Hall measurement under a reversible magnetic field of 1.5 T. The Hall carrier concentration *n*_H_ and Hall mobility μ_H_ were calculated from *n*_H_ = 1/*eR*_H_ and μ_H_ = σ*R*_H_, respectively.^43^ The Seebeck coefficient *S* was obtained from the slope of the Seebeck voltage versus temperature gradient in the temperature range of 0–5 K.^42^ The thermal conductivity κ was derived from κ = *dC*_p_α, where *d* is the mass density determined by the Archimedes method, *C*_p_ is the isobaric specific heat estimated from the Dulong–Petit limit, and α is the thermal diffusivity measured on a laser flash analyzer (LFA1000, Linseis) in a flowing helium atmosphere.^44^ The longitudinal ν_L_ and transverse ν_T_ sound velocities were gauged by using an ultrasonic pulse receiver (Olympus) equipped with an oscilloscope (Tektronix). The shear modulus *G* and bulk modulus *B* were calculated from the equations ν_T_ = (*G*/*d*)^1/2^ and ν_L_ = [(*B* + 4*G*/3)/*d*]^1/2^, respectively.

### Computational Details

All DFT calculations were performed using projector-augmented wave (PAW) pseudopotentials and Perdew–Burke–Ernzerhof generalized gradient approximation (GGA-PBE) exchange-correlation functional in the Vienna Ab initio Simulation Package (VASP).^45,46^ A plane-wave kinetic energy cutoff of 550 eV was used to truncate the basis in all DFT calculations. Geometry optimization was conducted on pristine ZnSb (eight Zn atoms and eight Sb atoms) and Zn_0.75_Cd_0.25_Sb, where doping was modeled by randomly substituting two Zn atoms with two Cd atoms. The composition ratio of Zn_0.75_Cd_0.25_Sb was chosen in the computational model to maintain a reasonable supercell size for computational efficiency while keeping the doping concentration close to that of our experimental composition of Zn_0.7_Cd_0.3_Sb. All structures were fully relaxed until the forces and energies reached the convergence criteria of 1 × 10^–4^ eV/Å and 1 × 10^–8^ eV, respectively. A Γ-centered Monkhorst-Pack scheme *k*-point mesh of 6 × 5 × 5 was adopted for the unit cell relaxation process, while the self-consistent force and energy calculations for phonon evaluation adopted a mesh of 3 × 2 × 2 on the supercell model.^47^ The electronic density of states (DOS) and band structure were evaluated using the modified Becke–Johnson (mBJ) potential, as it yields better agreement with experimental results.^48^ Postprocessing of electronic structure data was carried out using the VASPKIT package for extraction of electronic DOS and band structure data.^49^

Calculations of harmonic phonon dispersion relation, DOS, and group velocity were performed using the Phonopy package.^50^ 2 × 2 × 2 supercells of pristine ZnSb and Zn_0.75_Cd_0.25_Sb were employed in the finite displacement approach with a displacement amplitude of 0.01 Å to extract the second-order interatomic force constants of the systems.

## Results and Discussion

**Figure 1:**
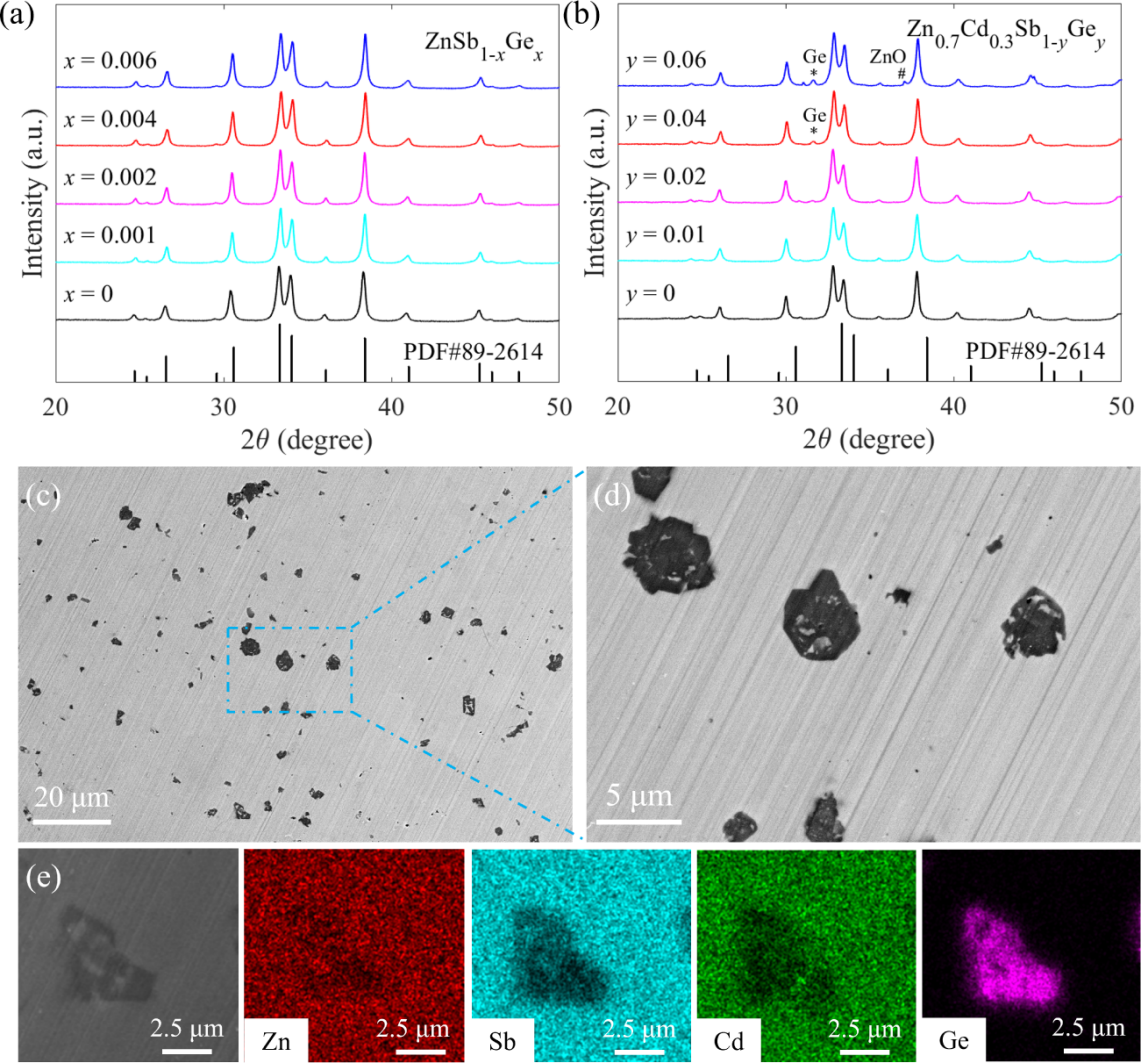
XRD patterns of (a) ZnSb_1–*x*_Ge*_x_* (*x* = 0, 0.001, 0.002, 0.004, and 0.006) and (b) Zn_0.7_Cd_0.3_Sb_1–*y*_Ge*_y_* (*y* = 0, 0.01, 0.02, 0.04, and 0.06) samples. BSE images of a polished surface for (c,d) Zn_0.7_Cd_0.3_Sb_0.96_Ge_0.04_. Elemental mappings of (e) Zn, Sb, Cd, and Ge for Zn_0.7_Cd_0.3_Sb_0.96_Ge_0.04_.

The X-ray diffraction (XRD) patterns of hot-pressed ZnSb_1–*x*_Ge*_x_* (*x* = 0, 0.001, 0.002, 0.004, and 0.006) and Zn_0.7_Cd_0.3_Sb_1–*y*_Ge*_y_* (*y* = 0, 0.01, 0.02, 0.04, and 0.06) samples are shown in Figure 1a and b, respectively. It is seen from Figure 1a that all diffraction peaks are well indexed to the ZnSb phase with an orthorhombic structure, and no obvious impurity peak is observed. This indicates that single-phase ZnSb_1–*x*_Ge*_x_* (*x* = 0, 0.001, 0.002, 0.004, and 0.006) samples are successfully synthesized. For Zn_0.7_Cd_0.3_Sb_1–*y*_Ge*_y_* (*y* = 0, 0.01, 0.02, 0.04, and 0.06) samples, as displayed in Figure 1b, all Bragg peaks show a systematic shift to the low-angle direction, which can be understood by the larger size of Cd than that of Zn. Moreover, when the Ge content reaches 0.04 and above, a secondary phase identified as Ge is detected. In addition to the Ge impurity phase, there is a small amount of ZnO in Zn_0.7_Cd_0.3_Sb_0.94_Ge_0.06_. The BSE images for a polished surface of hot-pressed Zn_0.7_Cd_0.3_Sb_0.96_Ge_0.04_ are presented in Figure 1c,d. As can be seen, numerous microscale precipitates are embedded in the matrix. According to elemental mapping results exhibited in Figure 1e, it is confirmed that the chemical composition of these microscale precipitates is Ge, which is in good agreement with XRD analysis.

**Figure 2:**
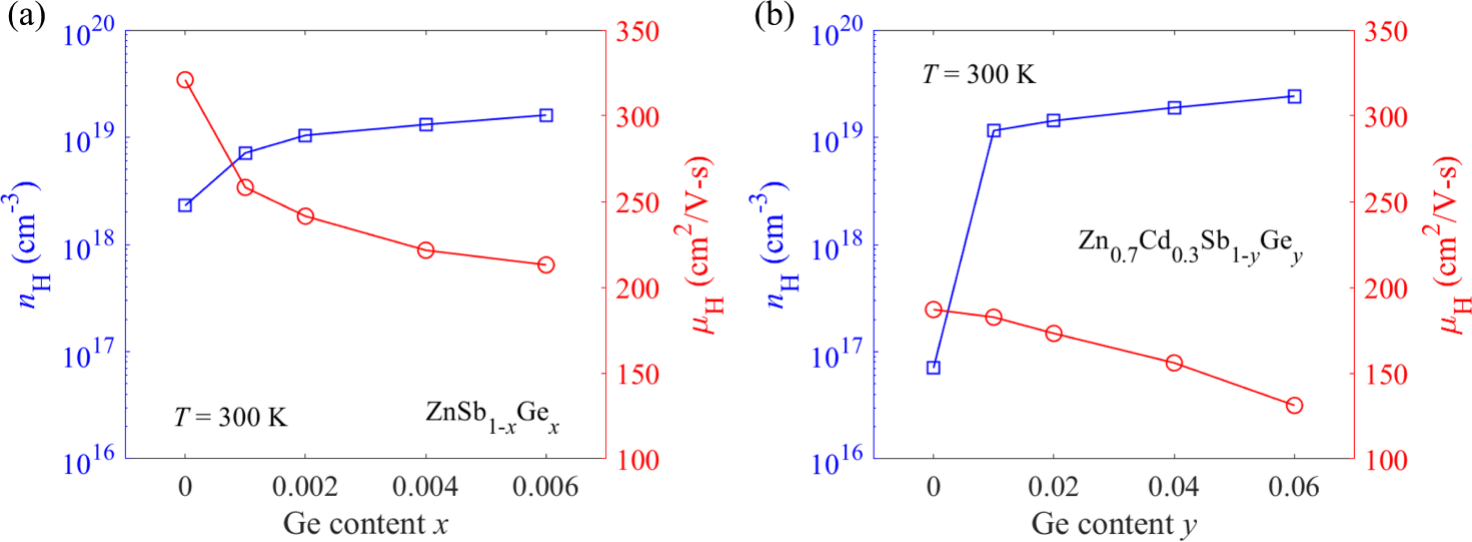
Room-temperature Hall carrier concentration and Hall mobility as a function of Ge content in (a) ZnSb_1–*x*_Ge*_x_* (*x* = 0, 0.001, 0.002, 0.004, and 0.006) and (b) Zn_0.7_Cd_0.3_Sb_1–*y*_Ge*_y_* (*y* = 0, 0.01, 0.02, 0.04, and 0.06) samples.

Pristine ZnSb is intrinsically a p-type semiconductor on account of its native Zn vacancies.^24^ It is seen from Figure 2a that the Hall carrier concentration of pristine ZnSb is 2.3 × 10^18^ cm^–3^ at room temperature, which is much lower than the optimal carrier concentration of 1.8 × 10^19^ cm^–3^.^51^ To increase the carrier concentration, Ge is chosen to substitute for Sb because Ge has one less valence electron than Sb. Furthermore, it is discovered that the formation energy of Ge_Sb_ substitutional defects is lower than that of Zn vacancies, which further shows the viability of Ge as an acceptor at the Sb site.^36^ Consequently, it is found from Figure 2a that room-temperature Hall carrier concentration is increased to 1.6 × 10^19^ cm^–3^ when Ge content is 0.006, which is approximately seven times that of pristine ZnSb. In the meantime, Hall mobility decreases from 321 cm^2^ V^–1^ s^–1^ for pristine ZnSb to 213 cm^2^ V^–1^ s^–1^ for ZnSb_0.994_Ge_0.006_ at room temperature. After introducing Cd into the Zn site, room-temperature Hall carrier concentration sharply falls to 7.0 × 10^16^ cm^–3^, and Hall mobility is simultaneously reduced to 187 cm^2^ V^–1^ s^–1^ at 300 K, as displayed in Figure 2b. Given a dramatic decrease in Hall carrier concentration after Cd alloying at the Zn site, a significant decline in Hall mobility can be ascribed to the strong point defect scattering of charge carriers. Besides, our DFT calculations reveal that the band gap becomes slightly smaller after Cd alloying at the Zn site, as depicted in Figure S1. By further incorporating Ge into Zn_0.7_Cd_0.3_Sb, room-temperature Hall carrier concentration is increased by about 2 orders of magnitude as opposed to that of Zn_0.7_Cd_0.3_Sb and is eventually raised to 2.4 × 10^19^ cm^–3^ when Ge content is 0.06. This again implies that substitution of Sb with Ge is effective in tuning the carrier concentration over a broad range. According to Figure S2a,b, it is seen that Hall mobility of all samples decays with temperature approximately via μ_H_ ∼ *T*^–1.5^ when temperature is above 450 K. This suggests that acoustic phonon scattering dominates the transport of charge carriers in this temperature range. However, when the temperature is below 400 K, the Hall mobility of Ge-doped Zn_0.7_Cd_0.3_Sb samples is nearly independent of temperature, indicating that charge carriers in Ge-doped Zn_0.7_Cd_0.3_Sb samples are mainly scattered by neutral impurities over this temperature range.

**Figure 3:**
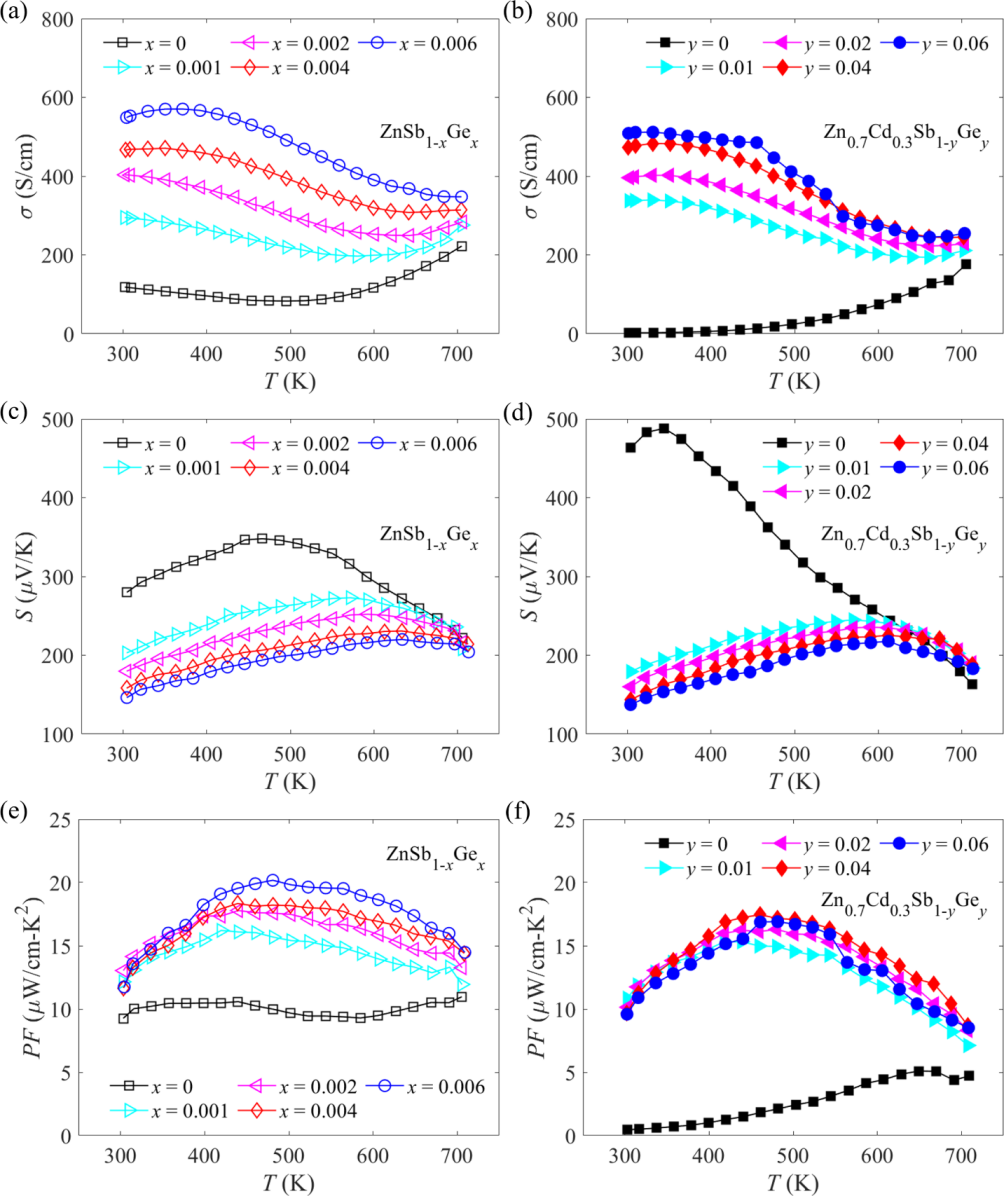
Temperature-dependent (a,b) electrical conductivities, (c,d) Seebeck coefficients, and (e,f) power factors of ZnSb_1–*x*_Ge*_x_* (*x* = 0, 0.001, 0.002, 0.004, and 0.006) and Zn_0.7_Cd_0.3_Sb_1–*y*_Ge*_y_* (*y* = 0, 0.01, 0.02, 0.04, and 0.06) samples.

Temperature-dependent electrical transport properties for all samples are displayed in Figure 3. The electrical conductivity of ZnSb_1–*x*_Ge*_x_* (*x* = 0, 0.001, 0.002, 0.004, and 0.006) and Zn_0.7_Cd_0.3_Sb_1–*y*_Ge*_y_* (*y* = 0, 0.01, 0.02, 0.04, and 0.06) samples as a function of temperature is presented in Figure 3a and b, respectively. As a result of increased carrier concentration, the electrical conductivity is improved from 118 S cm^–1^ for pristine ZnSb to 549 S cm^–1^ for ZnSb_0.994_Ge_0.006_ at room temperature, as shown in Figure 3a. After incorporating Cd into the Zn site, room-temperature electrical conductivity drastically decreases to 2 S cm^–1^, which can be attributed to the simultaneously reduced Hall carrier concentration and Hall mobility, as mentioned before. Additionally, the electrical conductivity of Zn_0.7_Cd_0.3_Sb increases with increasing temperature across the entire temperature range, which can be seen as indicative of semiconducting behavior. However, this semiconducting behavior transforms to a nearly metallic behavior after the substitution of Sb with Ge in Zn_0.7_Cd_0.3_Sb. More importantly, with the addition of Ge to Zn_0.7_Cd_0.3_Sb, room-temperature electrical conductivity is enhanced by about 2 orders of magnitude as compared with that of Zn_0.7_Cd_0.3_Sb and is finally increased to 509 S cm^–1^ when Ge content is 0.06.

The temperature dependences of Seebeck coefficients for ZnSb_1–*x*_Ge*_x_* (*x* = 0, 0.001, 0.002, 0.004, and 0.006) and Zn_0.7_Cd_0.3_Sb_1–*y*_Ge*_y_* (*y* = 0, 0.01, 0.02, 0.04, and 0.06) samples are exhibited in Figure 3c and d, respectively. A positive Seebeck coefficient indicates a p-type conduction for all samples, which is in compliance with the Hall measurement. Contrary to the variation of room-temperature electrical conductivity with Ge content, it is seen that the room-temperature Seebeck coefficient is reduced from 280 μV K^–1^ for pristine ZnSb to 146 μV K^–1^ for ZnSb_0.994_Ge_0.006_, and from 463 μV K^–1^ for Zn_0.7_Cd_0.3_Sb to 137 μV K^–1^ for Zn_0.7_Cd_0.3_Sb_0.94_Ge_0.06_. The temperature-dependent power factors for ZnSb_1–*x*_Ge*_x_* (*x* = 0, 0.001, 0.002, 0.004, and 0.006) and Zn_0.7_Cd_0.3_Sb_1–*y*_Ge*_y_* (*y* = 0, 0.01, 0.02, 0.04, and 0.06) samples are illustrated in Figure 3e and f, respectively. It is seen from Figure 3e that the peak power factor is largely enhanced from 11 μW cm^–1^ K^–2^ for pristine ZnSb at 705 K to 20 μW cm^–1^ K^–2^ for ZnSb_0.994_Ge_0.006_ at 480 K. Nevertheless, Cd alloying at the Zn site is detrimental to the electrical transport properties because the power factor of Zn_0.7_Cd_0.3_Sb is lower than that of pristine ZnSb over the entire temperature range. However, the substitution of Sb with Ge in Zn_0.7_Cd_0.3_Sb can promote a noticeable recovery in the power factor, as depicted in Figure 3f.

**Figure 4:**
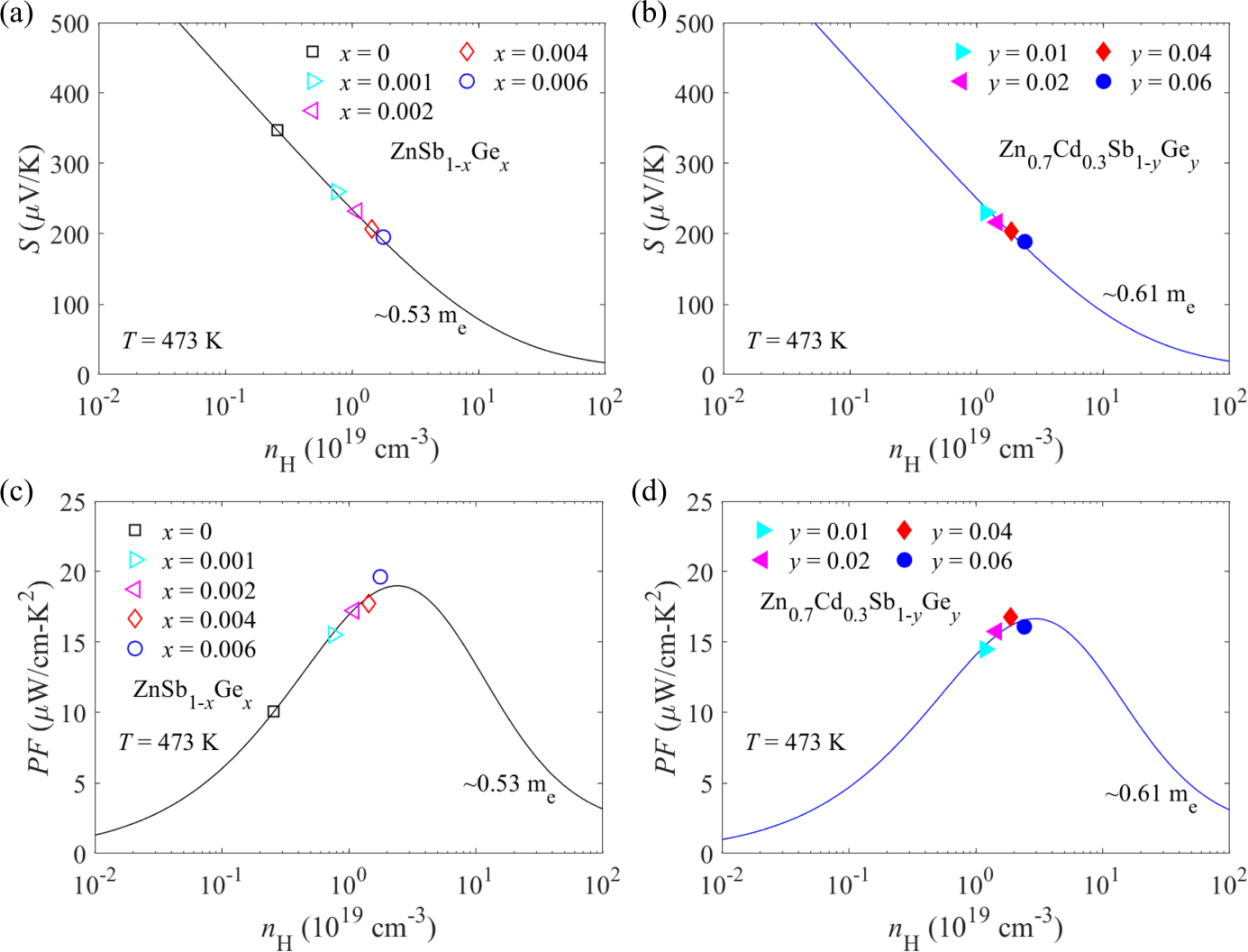
Hall carrier concentration-dependent (a,b) Seebeck coefficients and (c,d) power factors of ZnSb_1–*x*_Ge*_x_* (*x* = 0, 0.001, 0.002, 0.004, and 0.006) and Zn_0.7_Cd_0.3_Sb_1–*y*_Ge*_y_* (*y* = 0.01, 0.02, 0.04, and 0.06) samples at 473 K. The black and blue solid curves represent SPB models with DOS effective masses of ∼0.53*m*_e_ and ∼0.61*m*_e_, respectively.

The SPB model is utilized to gain more insights into the electrical transport properties of Ge-doped ZnSb and Ge-doped Zn_0.7_Cd_0.3_Sb samples under the assumption that charge carriers are dominantly scattered by acoustic phonons at 473 K.^52−54^ As shown in Figure 4a,b, the Pisarenko plot agrees well with our experimental results, which means that a trace amount of Ge has a limited effect on the DOS effective mass. Furthermore, it is found from Figure 4c,d that the theoretically predicted optimal carrier concentration for a maximum power factor at 473 K is achievable by adjusting the amount of Ge, suggesting that Ge is indeed a suitable dopant.

**Figure 5:**
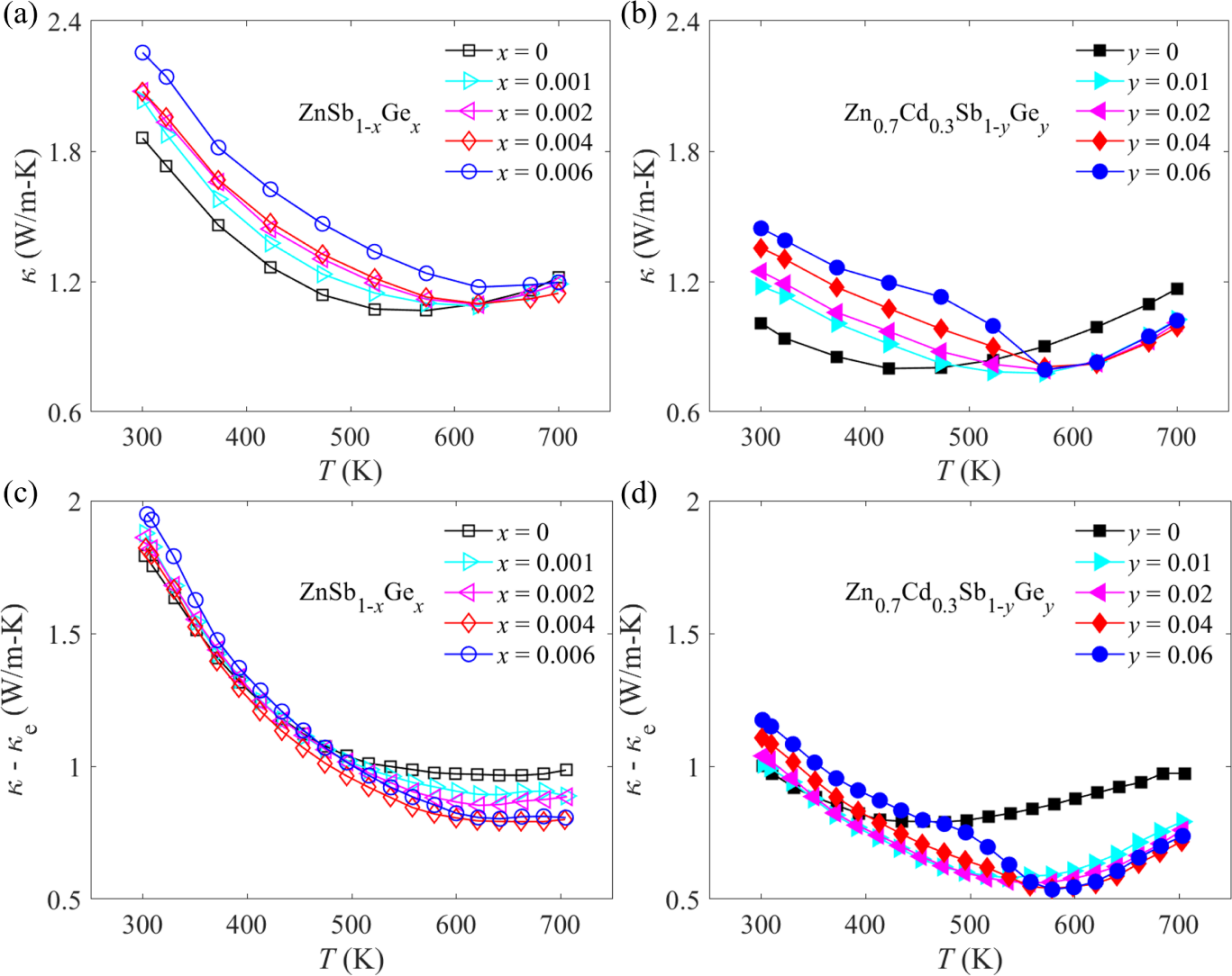
Temperature-dependent (a,b) thermal conductivities and (c,d) differences between total thermal conductivity and electronic thermal conductivity κ–κ_e_ of ZnSb_1–*x*_Ge*_x_* (*x* = 0, 0.001, 0.002, 0.004, and 0.006) and Zn_0.7_Cd_0.3_Sb_1–*y*_Ge*_y_* (*y* = 0, 0.01, 0.02, 0.04, and 0.06) samples.

Temperature-dependent thermal transport properties for all samples are illustrated in Figure 5. The total thermal conductivity κ is typically the sum of lattice thermal conductivity κ_L_ and electronic thermal conductivity κ_e_. However, it is reasonable to assume that the bipolar thermal conductivity κ_b_ is also included in the total thermal conductivity κ for this case because we notice that the total thermal conductivity for all samples appears to rise at high temperatures.^9,55^ Furthermore, it is found from Figure 5a that the total thermal conductivity of Ge-doped ZnSb samples increases with increasing Ge content when the temperature ranges from 300 to 573 K. This is because of the enhanced electronic thermal conductivity, as shown in Figure S3a. A similar phenomenon can be observed in Ge-doped Zn_0.7_Cd_0.3_Sb samples as well. The electronic thermal conductivity κ_e_ is proportional to the electrical conductivity through the Wiedemann–Franz relationship κ_e_ = *L*σ*T*, where *L* is the Lorenz number that can be derived from the measured Seebeck coefficient based on the SPB model.^55,56^ The sum of the lattice thermal conductivity κ_L_ and the bipolar thermal conductivity κ_b_ can be estimated by subtracting the electronic part κ_e_ from the total thermal conductivity κ. Since bipolar thermal conductivity κ_b_ at 300 K is usually negligible, the room-temperature value of κ–κ_e_ may be regarded as the lattice thermal conductivity κ_L_.^57,58^ It is seen from Figure 5c,d that the lattice thermal conductivity is largely reduced from 1.79 W m^–1^ K^–1^ for pristine ZnSb to 1 W m^–1^ K^–1^ for Zn_0.7_Cd_0.3_Sb at room temperature. This indicates that Cd alloying at the Zn site is very effective in suppressing phonon propagation.

A Callaway-type model is constructed to elucidate the origin of the suppressed lattice thermal conductivity through Cd alloying at the Zn site. In the framework of this model, the lattice thermal conductivity can be expressed as follows:^59,60^
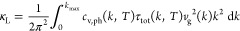
1

Here, *k*_max_ stands for the maximum phonon wave vector obtained from *k*_max_ = (6π^2^/*V*_avg_)^1/3^, where *V*_avg_ is the average volume per atom. The heat capacity of a single-phonon mode *c*_v,ph_ is given by  with *x* = ℏω/*k*_B_*T*, in which *k*_B_ is the Boltzmann constant, ℏ is the reduced Planck constant, and ω is the phonon frequency. Based on Matthiessen’s rule, the phonon relaxation time τ_tot_ can be calculated by considering the contributions from different scattering processes as follows:^19,20,61,62^
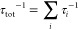
2

**Figure 6:**
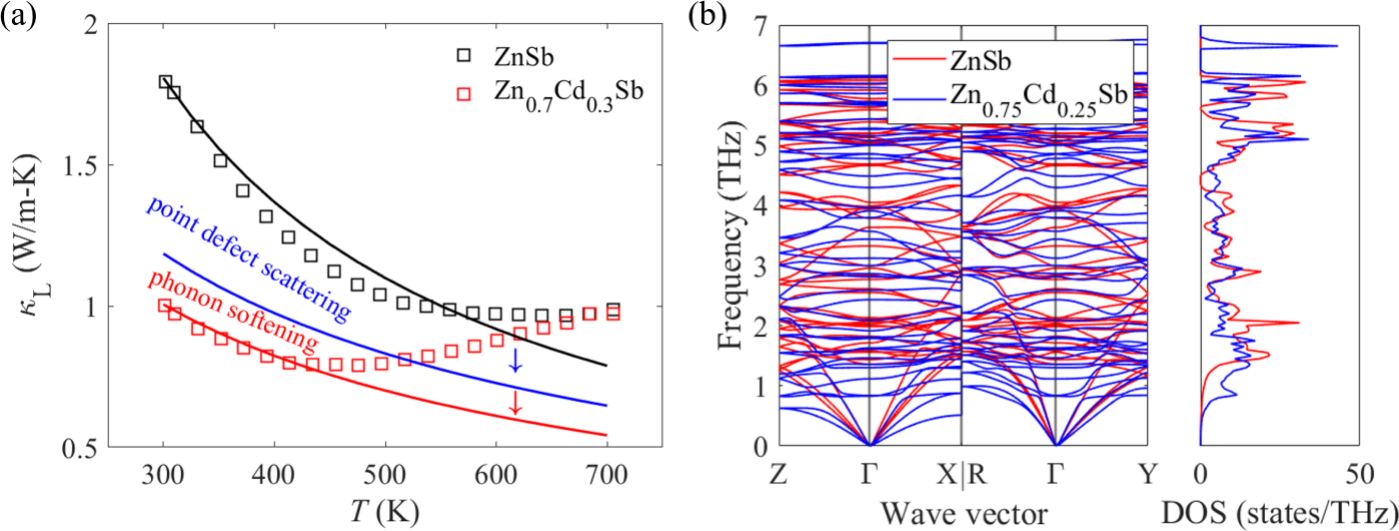
(a) Callaway-type model of lattice thermal conductivity under the Debye approximation. The reduction in κ_L_ from the black to blue curve is due to point defect scattering. The further reduction in κ_L_ from the blue to red curve is due to phonon softening. Experimental results for pristine ZnSb and Zn_0.7_Cd_0.3_Sb are displayed in black and red squares, respectively. (b) The calculated phonon dispersion relations for pristine ZnSb and Zn_0.75_Cd_0.25_Sb with the corresponding total phonon density of states (DOS).

The phonon group velocity *v*_g_ is described as *v*_g_ = δω/δ*k*. In the context of the Debye approximation, the phonon frequency ω is related to the phonon wave vector *k* via ω = *v*_s_*k*, where *v*_s_ denotes the average sound velocity. Therefore, the phonon group velocity in this model is identical to the average sound velocity under the Debye approximation. To unveil the role of point defect scattering in thermal transport, we calculate the phonon relaxation time for pristine ZnSb and Zn_0.7_Cd_0.3_Sb separately while the average sound velocity remains unchanged. Only Umklapp scattering, normal scattering, and grain boundary scattering are taken into consideration when evaluating the phonon relaxation time for pristine ZnSb, whereas point defect scattering as an extra source of thermal resistance is included in the case of Zn_0.7_Cd_0.3_Sb. As shown in Figure 6a, point defect scattering can result in a large decrease in the lattice thermal conductivity, especially at room temperature. This is due to mass and strain-field fluctuations caused by differences in atomic mass and size between Zn and Cd atoms.^17,63^ However, it is worth noting that such a drastic reduction in room-temperature lattice thermal conductivity can only be partly accounted for by point defect scattering. The results of sound velocity measurements as well as shear and bulk moduli for pristine ZnSb and Zn_0.7_Cd_0.3_Sb are listed in Table 1. Evidently, the drop in sound velocities and moduli after Cd alloying at the Zn site is a robust indicator of phonon softening. This necessitates the incorporation of phonon softening into this model to uncover its effect on thermal transport. After replacing the average sound velocity of pristine ZnSb with that of Zn_0.7_Cd_0.3_Sb, a further decrease in the lattice thermal conductivity, which is induced by phonon softening, is observed.^59,64^ It is noted that the discrepancy in the lattice thermal conductivity between experimental results and theoretical prediction at elevated temperatures can be explained by bipolar diffusion. To develop a deeper understanding of phonon softening, the phonon dispersion relations and phonon DOS for pristine ZnSb and Zn_0.75_Cd_0.25_Sb are calculated (Figure 6b). As can be seen, Cd alloying at the Zn site introduces low-frequency optical phonons at ∼1 THz and leads to the softening of acoustic phonons. The calculated group velocities of acoustic phonon modes for pristine ZnSb and Zn_0.75_Cd_0.25_Sb near the Γ point are listed in Table 2, which are consistent with the results of the sound velocity measurement listed in Table 1. Our findings show that Cd alloying at the Zn site can not only reduce the phonon relaxation time but also decrease the group velocity of acoustic phonon modes, leading to the suppressed lattice thermal conductivity.

**Table 1:** Longitudinal Sound Velocity ν_L_, Transverse Sound Velocity ν_T_, Average Sound Velocity ν_s_, Shear Modulus *G*, and Bulk Modulus *B* for Pristine ZnSb and Zn_0.7_Cd_0.3_Sb at Room Temperature

Sample	ν_L_ (m s^–1^)	ν_T_ (m s^–1^)	ν_s_ (m s^–1^)	*G* (GPa)	*B* (GPa)
Pristine ZnSb	4088	2206	2462	30.6	64.4
Zn_0.7_Cd_0.3_Sb	3744	2038	2273	26.9	54.9

**Table 2:** Calculated Group Velocities (m s^–1^) of Acoustic Phonon Modes for Pristine ZnSb and Zn_0.75_Cd_0.25_Sb near the Γ Point

	Pristine ZnSb		Zn_0.75_Cd_0.25_Sb	
	Γ–*Z*	Γ–*X*	Γ–*Z*	Γ–*X*
TA	2439	2326	1585	1737
TA′	2841	2562	2108	2261
LA	4227	3987	4118	3823

**Figure 7:**
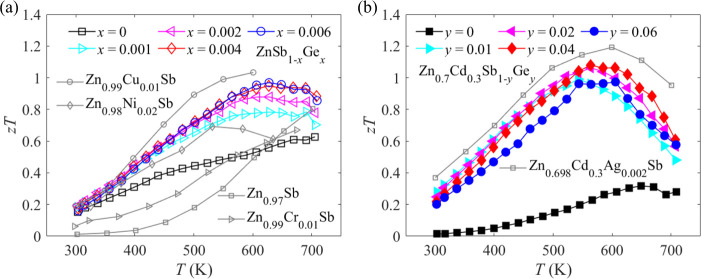
Temperature-dependent *zT* values of (a) ZnSb_1–*x*_Ge*_x_* (*x* = 0, 0.001, 0.002, 0.004, and 0.006) and (b) Zn_0.7_Cd_0.3_Sb_1–*y*_Ge*_y_* (*y* = 0, 0.01, 0.02, 0.04, and 0.06) samples. *zT* values previously reported for other ZnSb-based thermoelectric materials are included for comparison.^24,31,65−67^

The temperature dependence of *zT* values for all samples is shown in Figure 7. The peak *zT* value of pristine ZnSb is ∼0.63 at 700 K. Benefiting from remarkable improvement in power factor and significant suppression of lattice thermal conductivity, the peak *zT* value is enhanced to ∼1.08 at 564 K for Zn_0.7_Cd_0.3_Sb_0.96_Ge_0.04_. It can be seen from Figure 7a,b that the peak *zT* value achieved in this work is comparable to those previously reported for other ZnSb-based thermoelectric materials.

## Conclusion

In summary, TE properties of p-type ZnSb_1–*x*_Ge*_x_* (*x* = 0, 0.001, 0.002, 0.004, and 0.006) and Zn_0.7_Cd_0.3_Sb_1–*y*_Ge*_y_* (*y* = 0, 0.01, 0.02, 0.04, and 0.06) samples are thoroughly investigated in this work. Ge doping at the Sb site can enable carrier concentration optimization and thus considerable enhancement of the electrical transport properties. In addition, Cd alloying at the Zn site can significantly suppress the lattice thermal conductivity owing to point defect scattering and phonon softening. By virtue of a synergistic improvement in electrical and thermal transport properties, a peak *zT* value of ∼1.08 is achieved in Zn_0.7_Cd_0.3_Sb_0.96_Ge_0.04_ at 564 K, confirming that ZnSb is a promising TE material.

## Supporting Information Available

Calculation of phonon relaxation time for pristine ZnSb and Zn_0.7_Cd_0.3_Sb; actual chemical compositions of Ge-doped Zn_0.7_Cd_0.3_Sb samples; electronic band structures and densities of states (DOS) for pristine ZnSb and Zn_0.75_Cd_0.25_Sb; temperature-dependent Hall mobility and electronic thermal conductivity for all samples (PDF)
