# Supplementary material for: Point Defect Scattering and Phonon Softening for Achieving High Thermoelectric Performance in p-Type ZnSb with Optimal Carrier Concentration
Source: ACS Appl Mater Interfaces. 2025 Mar 10;17(11):17036–44. doi: 10.1021/acsami.4c21670 (PMC11931488; doi:10.1021/acsami.4c21670)
Supplement: Supplementary file 1 — am4c21670_si_001.pdf [file am4c21670_si_001.pdf]

Supporting Information

Point defect scattering and phonon softening for achieving high thermoelectric performance in p-type ZnSb with optimal carrier concentration

Dongyi Shen[1], Siu Ting Tai[1], Kejia Liu[1], Wenxuan Wang[1], Haiqi Li[1], Vaskuri C. S. Theja[1], Chen Chen[2, *], Yue Chen[1, *]

[1]*Department of Mechanical Engineering, The University of Hong Kong, Pokfulam Road, Hong Kong SAR, China, E-mail:* yuechen@hku.hk

[2]*School of Physical Sciences, Great Bay University, Dongguan, Guangdong, 523000, China, E-mail:* ccmldn@gbu.edu.cn

The calculation of phonon relaxation time for pristine ZnSb and $Zn_{0.7}Cd_{0.3}Sb$

The phonon relaxation time for Umklapp and normal scatterings is expressed as below:[1, 2]

$$\tau_U^{-1} + \tau_N^{-1} = A_N \frac{2}{\left(6\pi^2\right)^{1/3}} \frac{k_B V_{avg}^{1/3} \gamma^2 \omega^2 T}{M_{avg} v_s^3}, \tag{S1}$$

where A_N is an additional factor fitted based on the experimental data, V_{avg} is the average volume per atom, γ is the Grüneisen parameter, ω is the phonon frequency, M_{avg} is the average mass per atom, and v_s is the average sound velocity.

The average sound velocity v_s can be calculated from transverse v_T and longitudinal v_L sound velocities as below:[3, 4]

$$v_s = \left[\frac{1}{3} \left(\frac{1}{v_L^3} + \frac{2}{v_T^3} \right) \right]^{-1/3}. \tag{S2}$$

Grüneisen parameter γ can be written as the following equation:[3, 4]

$$\gamma = \frac{3}{2} \left(\frac{1 + \upsilon_p}{2 - 3\upsilon_p} \right), \tag{S3}$$

where υ_p is the Poisson's ratio derived from $\upsilon_p = \frac{1-2(v_T/v_L)^2}{2-2(v_T/v_L)^2}$.

The phonon relaxation time for grain boundary scattering is described as[1, 2]

$$\tau_{GB}^{-1} = \frac{v_s}{L_{grain}}, \tag{S4}$$

in which L_{grain} is the average grain size.

The phonon relaxation time for point defect scattering is given by[1, 2, 5]

$$\tau_{PD}^{-1} = \frac{V_{avg} \omega^4}{4\pi v_s^3} \Gamma. \tag{S5}$$

The disorder scattering parameter Γ can be obtained from the relation as below:[5, 6]

$$\Gamma = \Gamma_M + \Gamma_S. \tag{S6}$$

In the case of two different atoms at each of the ith crystallographic sublattice, the disorder scattering parameter due to mass fluctuation Γ_M is defined as[6]

$$\Gamma_M = \frac{\sum_{i=1}^{n} c_i \left(\frac{\overline{M_i}}{\overline{\overline{M}}}\right)^2 f_i^1 f_i^2 \left(\frac{M_i^1 - M_i^2}{\overline{M_i}}\right)^2}{\left(\sum_{i=1}^{n} c_i\right)}. \quad \text{(S7)}$$

The disorder scattering parameter due to strain-field fluctuation Γ_S is described as[6]

$$\Gamma_S = \frac{\sum_{i=1}^{n} c_i \left(\frac{\overline{M_i}}{\overline{\overline{M}}}\right)^2 f_i^1 f_i^2 \varepsilon \left(\frac{r_i^1 - r_i^2}{\overline{r_i}}\right)^2}{\left(\sum_{i=1}^{n} c_i\right)}. \quad \text{(S8)}$$

Here, c_i is the relative degeneracy of the ith crystallographic sublattice, f_i^1 and f_i^2 are fractional occupations, M_i^1 and M_i^2 are the masses for each atom in the ith crystallographic sublattice, and r_i^1 and r_i^2 are the radii for each atom in the ith crystallographic sublattice. $\overline{M_i}$ is the average mass of atoms in the ith crystallographic sublattice, which is defined as $\overline{M_i} = f_i^1 M_i^1 + f_i^2 M_i^2$. $\overline{r_i}$ is the average radius of atoms in the ith crystallographic sublattice, which is defined as $\overline{r_i} = f_i^1 r_i^1 + f_i^2 r_i^2$. $\overline{\overline{M}}$ is the average atomic mass of the compound. ε is a phenomenological adjustable parameter calculated from the formula $\varepsilon = \frac{2}{9}\left[\frac{6.4\times\gamma(1+\upsilon_p)}{1-\upsilon_p}\right]^2$.[4, 6]

Table S1 Parameters used in Callaway-type model to calculate the lattice thermal conductivity of pristine ZnSb.

Parameter	Symbol (unit)	Value
Additional factor	A_N	6.32
Average volume per atom	V_{avg} (m^3)	2.44×10^{-29}
Average mass per atom	M_{avg} (kg)	1.55×10^{-25}
Average sound velocity	v_s (m s^{-1})	2462
Poisson's ratio	υ_p	0.29
Grüneisen parameter	γ	1.74
Average grain size	L_{grain} (m)	3.5×10^{-5}
Disorder scattering parameter	Γ	0
Phenomenological adjustable parameter	ε	92

Debye temperature	θ_D (K)	253

Table S2 Parameters used in Callaway-type model to calculate the lattice thermal conductivity of $Zn_{0.7}Cd_{0.3}Sb$.

Parameter	Symbol (unit)	Value
Additional factor	A_N	5.75
Average volume per atom	V_{avg} (m^3)	2.56×10^{-29}
Average mass per atom	M_{avg} (kg)	1.67×10^{-25}
Average sound velocity	v_s ($m\ s^{-1}$)	2273
Poisson's ratio	υ_p	0.29
Gruneisen parameter	γ	1.71
Average grain size	L_{grain} (m)	3.5×10^{-5}
Disorder scattering parameter	Γ	0.11
Phenomenological adjustable parameter	ε	87
Debye temperature	θ_D (K)	230

Table S3 The actual chemical compositions of $Zn_{0.7}Cd_{0.3}Sb_{1-y}Ge_y$ (y = 0.01, 0.02, 0.04 and 0.06) samples determined by EDS.

Nominal chemical composition	Actual chemical composition
$Zn_{0.7}Cd_{0.3}Sb_{0.99}Ge_{0.01}$	$Zn_{0.700}Cd_{0.301}Sb_{0.99}Ge_{0.006}$
$Zn_{0.7}Cd_{0.3}Sb_{0.98}Ge_{0.02}$	$Zn_{0.711}Cd_{0.281}Sb_{0.98}Ge_{0.006}$
$Zn_{0.7}Cd_{0.3}Sb_{0.96}Ge_{0.04}$	$Zn_{0.701}Cd_{0.281}Sb_{0.96}Ge_{0.009}$
$Zn_{0.7}Cd_{0.3}Sb_{0.94}Ge_{0.06}$	$Zn_{0.686}Cd_{0.286}Sb_{0.94}Ge_{0.012}$

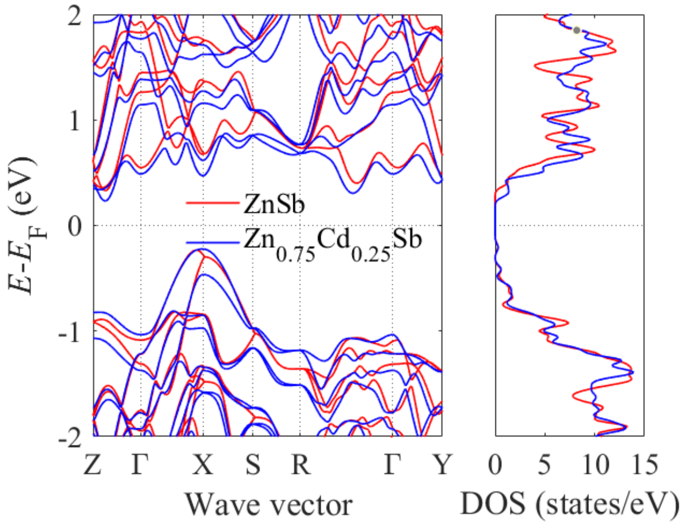

Figure S1 Electronic band structures and densities of states (DOS) for pristine ZnSb and $Zn_{0.75}Cd_{0.25}Sb$.

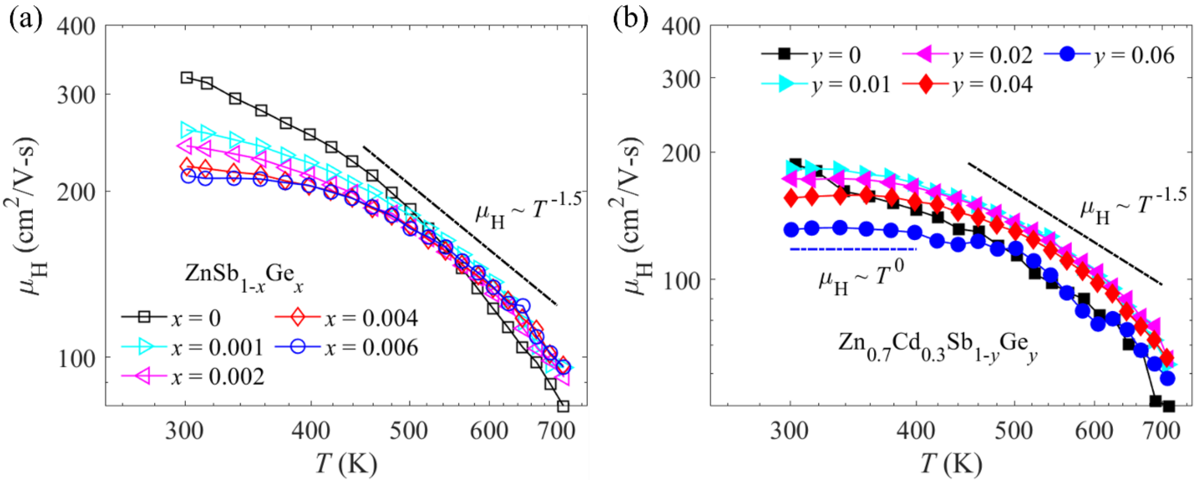

Figure S2 Temperature-dependent Hall mobility of (a) $ZnSb_{1-x}Ge_x$ (x = 0, 0.001, 0.002, 0.004 and 0.006) and (b) $Zn_{0.7}Cd_{0.3}Sb_{1-y}Ge_y$ (y = 0, 0.01, 0.02, 0.04 and 0.06) samples.

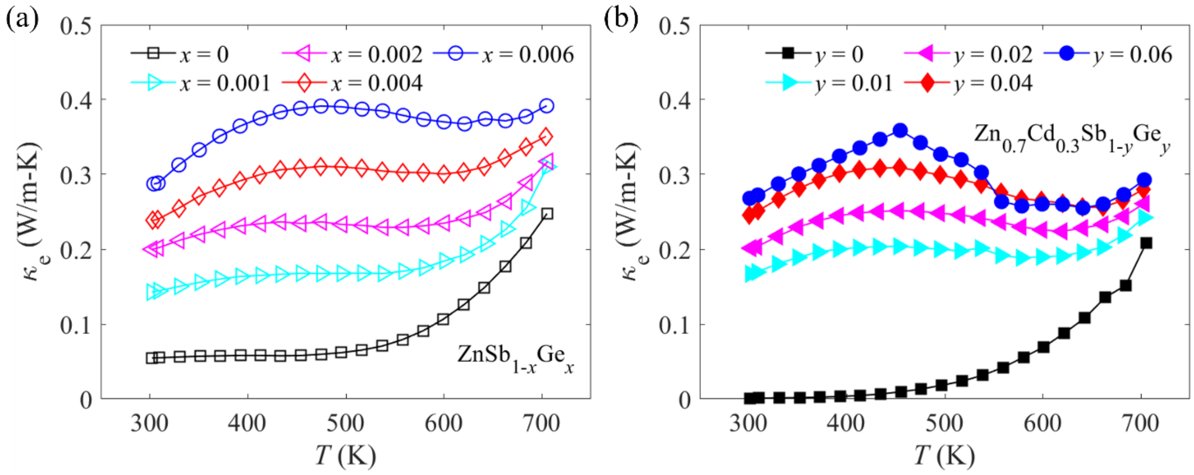

Figure S3 Temperature-dependent electronic thermal conductivity of (a) $ZnSb_{1-x}Ge_x$ (x = 0, 0.001, 0.002, 0.004 and 0.006) and (b) $Zn_{0.7}Cd_{0.3}Sb_{1-y}Ge_y$ (y = 0, 0.01, 0.02, 0.04 and 0.06) samples.

References

(1) Chen, C.; Shen, D.; Xia, C.; Zhang, Z.; Wang, W.; Zhang, Q.; Chen, Y. Integrating band engineering with point defect scattering for high thermoelectric performance in $Bi_2Si_2Te_6$. *Chem. Eng. J.* **2022**, *441*, 135968.

(2) Bao, X.; Liu, K.; Xue, W.; Yao, H.; Ma, X.; Li, X.; Ye, S.; Cao, F.; Mao, J.; Zhang, Q. Multiscale phonon scattering for ultra-low thermal conductivity in co-doped ZrCoBi half-Heusler. *Adv. Funct. Mater.* **2024**, *34* (41), 2404279.

(3) Xie, H.; Wang, H.; Pei, Y.; Fu, C.; Liu, X.; Snyder, G. J.; Zhao, X.; Zhu, T. Beneficial contribution of alloy disorder to electron and phonon transport in half-Heusler thermoelectric materials. *Adv. Funct. Mater.* **2013**, *23* (41), 5123-5130.

(4) Hu, C.; Xia, K.; Chen, X.; Zhao, X.; Zhu, T. Transport mechanisms and property optimization of p-type (Zr, Hf) CoSb half-Heusler thermoelectric materials. *Mater. Today Phys.* **2018**, *7*, 69-76.

(5) Wang, X.; Yao, H.; Yin, L.; Xue, W.; Zhang, Z.; Duan, S.; Chen, L.; Chen, C.; Sui, J.; Liu, X. Band modulation and strain fluctuation for realizing high average zT in GeTe. *Adv. Energy Mater.* **2022**, *12* (26), 2201043.

(6) Yang, J.; Meisner, G.; Chen, L. Strain field fluctuation effects on lattice thermal conductivity of ZrNiSn-based thermoelectric compounds. *Appl. Phys. Lett.* **2004**, *85* (7), 1140-1142.
